# Human T-cell leukemia virus type 1 is associated with dysthyroidism in the French Amazon

**DOI:** 10.3389/fcimb.2023.1164526

**Published:** 2023-05-24

**Authors:** Julia Dugardin, Magalie Demar, Nezha Hafsi, Hakim Amroun, Jean-Markens Aurelus, Kinan Drak Alsibai, André Ntoutoum, Florin Santa, Mathieu Nacher, Nadia Sabbah

**Affiliations:** ^1^ Department of Endocrinology and Metabolic Diseases, Cayenne Hospital Center, Cayenne, French Guiana; ^2^ Laboratory of Parasitology-Mycology (LHUPM), Cayenne Hospital Center, Cayenne, French Guiana; ^3^ Department of Surgery, Cayenne Hospital Center, Cayenne, French Guiana; ^4^ Department of Pathology and Center of Biological Resources (CRB Amazonie), Cayenne Hospital Center, Cayenne, French Guiana; ^5^ Department of Internal Diseases, Cayenne Hospital Center, Cayenne, French Guiana; ^6^ Clinical Investigation Center Antilles French Guiana (CIC INSERM 1424), Cayenne Hospital Center, Cayenne, French Guiana

**Keywords:** HTLV- 1, hypothyroidism, hyperthyroidism, thyroiditis, French Guiana, TSH (thyroid stimulating hormone)

## Abstract

**Background:**

Human T-cell leukemia virus type 1 (HTLV-1) is a retrovirus known to cause two major diseases: adult T-cell leukemia/lymphoma and a progressive neuromyelopathy—tropical spastic paraparesis. Many viruses may be involved in the pathogenesis of thyroiditis; however, few studies have focused on the role of HTLV-1. We aimed to investigate the association between HTLV-1 and biological thyroid dysfunction.

**Methods:**

We included 357 patients with a positive HTLV-1 serology and thyroid-stimulating hormone assay data between 2012 and 2021 in a hospital in French Guiana; we compared the prevalence of hypothyroidism and hyperthyroidism in this group with that in an HTLV-1-negative control group (722 persons) matched for sex and age.

**Results:**

The prevalence of hypothyroidism and hyperthyroidism in patients with HTLV-1 infection was significantly higher than that in the control group (11% versus 3.2% and 11.3% versus 2.3%, respectively; *p* < 0.001).

**Conclusion:**

Our study shows, for the first time, the association between HTLV-1 and dysthyroidism in a large sample, suggesting that thyroid function exploration should be systematically implemented in this population as this may have an impact on therapeutic management.

## Introduction

Human T-cell leukemia virus type 1 (HTLV-1) is a retrovirus that induces the transformation and clonal expansion of T cells, mainly CD4, but also CD8 T cells; it can also infect other cells in different tissues (Gross and Thoma-Kress, 2016; Mozhgani et al., 2022). Infection occurs mainly through cell–cell contact (Carpentier et al., 2015). Recent studies have estimated the worldwide HTLV-1-infected population to be between 5 and 10 million (Gessain and Cassar, 2012). HTLV-1 is not ubiquitous but highly endemic in certain regions of the world: southeast Japan, Latin America, the Caribbean basin, and intertropical Africa, with a few outbreaks being reported in Australo-Melanesia, the Middle East, and Romania (Gessain and Cassar, 2012). The three modes of transmission include vertical transmission from mother to child occurring mainly during prolonged breastfeeding, sexual transmission, and parenteral transmission. In endemic areas, HTLV-1 infections are strongly associated with age and sex with the number of infections increasing with age and being more common in women. The diagnosis is based on the detection of anti-HTLV-1 antibodies in the serum.

The seriousness of the disease is attributable to two major types of complications. HTLV-1 infections can progress to adult T-cell leukemia/lymphoma (ATLL), a malignant proliferation of T cells with a poor prognosis and a median survival of 6 months in its acute form (Shimoyama, 1991; Gessain and Cassar, 2012; Forlani et al., 2021). The second most frequently encountered disorder is a progressive neuromyelopathy: tropical spastic paraparesis/HTLV-1 associated myelopathy (HAM/TSP) (Kira et al., 1992; Forlani et al., 2021).

However, the virus can affect different organs and cause various other symptoms. Other conditions include pulmonary alveolitis; infectious dermatitis; chronic arthropathy; and autoimmune diseases such as uveitis, polymyositis, and Sjögren syndrome (Sugimoto et al., 1987; Kawai et al., 1996; Watanabe et al., 1997; Ramezani et al., 2022).

Associations between thyroiditis and viral infection have been described for retroviruses (HTLV-1, HFV, HIV, and SV40) in Graves’ disease, and for HTLV-1, enterovirus, rubella, mumps virus, HSV, EBV, and parvovirus in Hashimoto’s thyroiditis (Desailloud and Hober, 2009). Infectious agents of hepatitis C are one of the most common viral etiologies (Nazary et al., 2021). The association between thyroid autoimmune diseases and HTLV-1 has been sporadically described in the literature, often in small cohorts from Japan (Kawai et al., 1992b; Mizokami et al., 1994; Kawai et al., 1995; Yanagawa et al., 1995; Akamine et al., 1996; Kubonishi et al., 1997). Few studies have found a significantly higher prevalence of HTLV-1-positive patients in populations of patients with Hashimoto thyroiditis or Graves’ disease (Kawai et al., 1992a; Mizokami et al., 1994).

French Guiana is a French territory located on the north coast of South America between Brazil and Suriname; 95% of its 83,534 km²-wide surface is covered by primary forest. HTLV-1 is endemic in this region; however, its prevalence varies among ethnic groups. In this regard, there is a marked difference in the prevalence between Maroons (descendants of runaway slaves) and Wayana Amerindians (10.3% and 0%, respectively) (Gessain et al., 1984). Another study in different ethnic groups of African ancestry reported an HTLV-1 seroprevalence of 6.7% (Plancoulaine et al., 1998). Seroprevalence increased with age and was significantly higher in women, reaching 40% in Maroon women aged >50 years. In 1992, a study found an HTLV-1 seroprevalence of 4.4% in pregnant women in western French Guiana, where Maroon ethnic groups predominate (Carles et al., 2004). The description of an association between HTLV-1 infection and hyperthyroidism of unknown etiology in French Guiana led us to expand our investigation to compare the prevalence of hypothyroidism and hyperthyroidism in patients with and without HTLV-1 infection (Amelie et al., 2020).

## Materials and methods

### Study type

In this cross-sectional study, we compared the prevalence of hyperthyroidism and hypothyroidism in HTLV-1-positive and HTLV-1-negative patients; additionally, we compared the prevalence of these conditions between groups by age and sex.

### Data collection

Data were collected from computerized patient files and anonymized. We collected the data of patients with HTLV-1-positive serologies by ELISA (enzyme-linked immunosorbent assay) with confirmation of HTLV-1 infection by Western blot or PCR (polymerase chain reaction). Serum samples from patients were tested for the detection of HTLV-1/2 antibodies using a CMIA (chemiluminescent microparticle immunoassay) method (ARCHITECT rHTLV-I/II, Abbott Laboratories, Wiesbaden, Germany) following the manufacturer’s instructions. Patients for whom the first screening result was positive benefitted from a confirmatory assay with a Line immune assay test (INNO-LIA HTLVI/II Score, FURIJEBIO Europe, Belgium). This confirmation was performed at the Cayenne Hospital laboratory between 2012 and 2021; the samples were taken from adult patients who were hospitalized in Cayenne Hospital or in the remote health centers (providing care in the most isolated areas of the Amazonian forest), or from outpatients with at least one thyroid-stimulating hormone (TSH) assay performed by the hospital laboratory. In the case of patients with confirmed HTLV-1 infections, we collected data on TSH (laboratory value, 0.4–4.2 mIU/ml), along with the date of sample collection. As we could not confirm the TSH values, we excluded patients for whom TSH measurement was performed in a private laboratory and documented on the computerized patient files.

In cases of HTLV-1 positivity, patients are routinely tested for Epstein–Barr virus and hepatitis C. Concerning the rubeola virus, we only test pregnant women (excluded from the study) and parvovirus B19 is only tested if there are clinical symptoms. In our study, none of the patients had associated hepatitis C or EBV serology and none of the patients were tested for parvovirus B19 or rubella.

Furthermore, we collected data on age, sex, and place of residence classified into savannah plateau, coastal center, eastern French Guiana, and western French Guiana, and missing information.

### Control population

Controls were defined as patients who were hospitalized and had both negative HTLV serology and a thyroid checkup during hospitalization. Patients were randomly selected from the laboratory databases of clinical wards unrelated to thyroid disease management from 2012 to 2021. The maximum interval between requests for HTLV serology and thyroid checkups was less than 1 month. In addition, only patients with a single thyroid checkup request during this period were selected to ensure the absence of thyroid pathology and, therefore, medical follow-up.

Thus, the controls were individually matched to the cases by sex and age (1:2 for women and 1:3 for men) using the Mahalanobis optimal algorithm (XLSTAT 2022.3.1.1344).

### Definitions of biological hypothyroidism and hyperthyroidism

A TSH level of less than 0.4 mIU/L was defined as hyperthyroidism, and a TSH level ≥4.2 mIU/L was defined as hypothyroidism. As TSH assays were performed in most cases as a screening test and not as a diagnostic test, free thyroid hormones (T4L and T3L) were mostly unmeasured; therefore, we could not differentiate between subclinical and confirmed dysthyroidism.

### Inclusion and exclusion criteria

The inclusion criteria were as follows:

- Patients over 18 years of age who underwent at least one serological analysis for HTLV-1 and one TSH measurement at the Cayenne Hospital Laboratory or a remote health center
- Patients who did not decline participation after being informed by letters and posters displayed in the hospital laboratory

**Figure 1:**
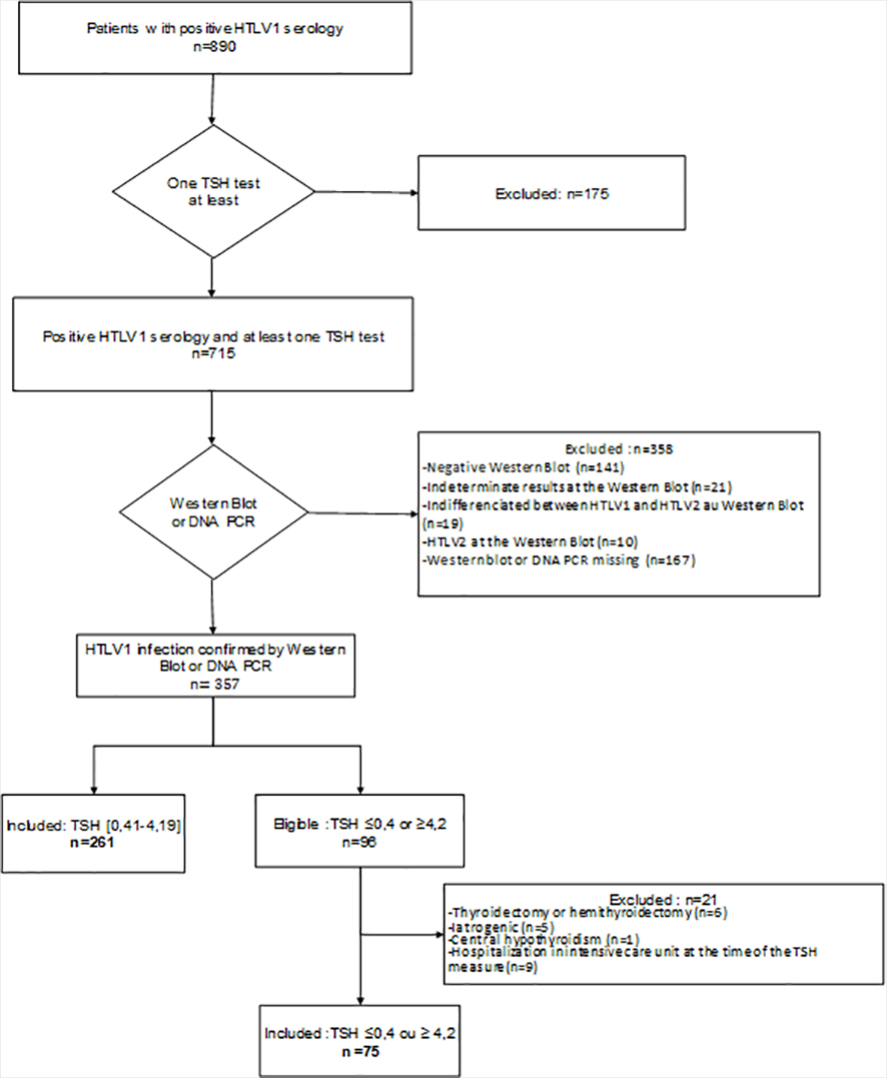
Flowchart of the HTLV-1 population.

**Figure 2:**
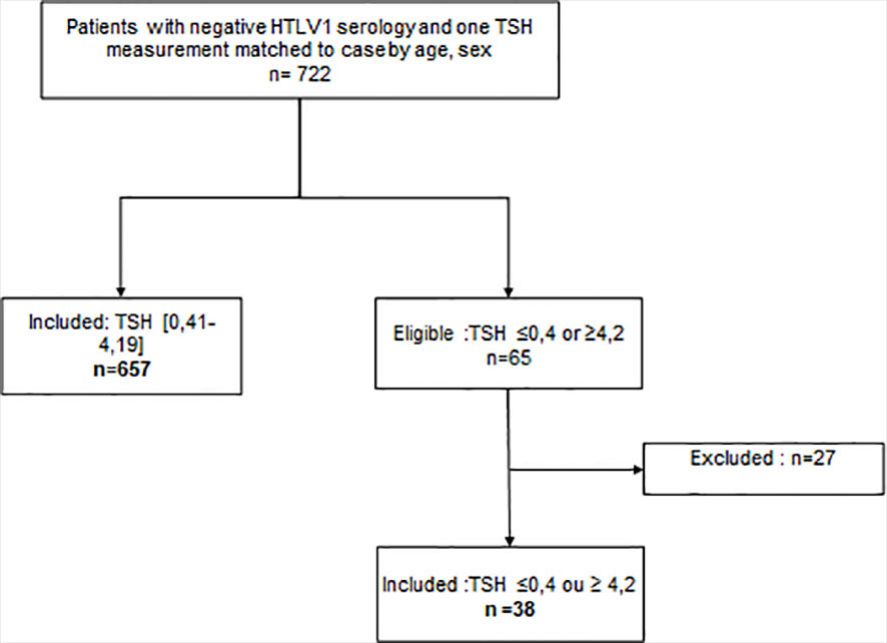
Flowchart of the control population.

The exclusion criteria were as follows (**Figures 1** and **2**): patients with known dysthyroidism; those who had undergone thyroidectomy, hemithyroidectomy, or radioactive iodine therapy; pregnant women; patients on treatments that may cause dysthyroidism (interferon, iodine, lithium) at the time of sampling; patients who were hospitalized in the intensive care unit at the time of collection; and, patients with central hypothyroidism.

### Statistical analysis

The statistical analysis was performed using the STATA 16 software (STATA-CORP^®^) (StataCorpLLC, Lakeway Drive College Station, Texas, USA). A descriptive analysis of the study population and clinical data was performed. Quantitative data are expressed as means and standard deviations for continuous variables, and prevalence data are expressed as frequencies and percentages. Age was categorized as ≤30 years, 30–65 years, and >65 years. The proportions of HTLV-1-positive patients with and without hyperthyroidism and hypothyroidism were compared using crude odds ratios and the *χ* ^2^ test. Furthermore, we used two unconditional multiple logistic regression models to control for age, sex, and residence location (there may be ethnic or iodine input differences among the different parts of French Guiana): one with hyperthyroidism as the dependent variable and the other with hypothyroidism as the dependent variable. Because a matched analysis does not necessarily control for confounders, an unmatched analysis was performed to control for two important confounders: age and sex. To quantify the effect size with this cross-sectional data, we computed the risk difference between HTLV-1-positive and -negative patients based on age, sex, and residence location, using inverse probability weighting. Statistical *p-*values <0.05 were considered significant.

### Ethical and regulatory issues

The study protocol was approved by the Ethics Committee and the Institutional Review Board of French Guiana with number of approvals. All the patients were informed by letter that their anonymized data would be used for the research, and none of them opposed it. Thereafter, all the records were anonymized. In accordance with the French Data Protection Act and the General Data Protection Regulation, data processing was subject to a data protection impact analysis, entry into the hospital’s data processing registry, and a declaration of compliance to the MR004.

## Results

### Description of the population

The description of the population is summarized in **Table 1**.

**Table 1:** Description of the study population.

Variables	HTLV-1 population *N* (%)	Control population *N* (%)
Women	227 (67.56)	379 (54.53)
Men	109 (32.44)	316 (45.47)
Average age (years)	63.94	55.79
Age category 18–30 years	17 (5.06)	57 (8.20)
Age category 30–65 years	137 (40.48)	421(60.57)
Age category over 65	182 (54.16)	217 (31.22)
Residence		
Savannah Plateau	8 (2.38)	16 (2.30)
Eastern French Guiana	7 (2.08)	18 (2.59)
Western French Guiana	57 (16.96)	26 (3.74)
Coastal Center	211 (62.80)	635 (91.37)
Unknown	53 (15.77)	0

There were 336 patients with a positive serology for HTLV-1 and 695 controls. There were 227 women and 109 men in the HTLV-1 group (67.56% and 32.44%, respectively), with an average age of 64 years. Furthermore, 54% and 5% of the patients were >65 and <30 years old, respectively. The patients were primarily from the coastal regions of French Guiana.

There were significantly more HTLV-1-infected women and proportionally more patients in regions with a high prevalence of HTLV-1 seropositivity, notably western French Guiana (16.96%).

### Association between abnormal TSH and HTLV-1

**Table 2** shows the significant association between HTLV-1 infection and hyperthyroidism before and after adjusting for age, sex, and residential location. The prevalence of hyperthyroidism was 11.3% and 2.3% in the HTLV-1 and control groups, respectively. The overall odds ratios of hyperthyroidism in patients with HTLV-1 infection before and after adjustment were 5.1 and 6.8, respectively (*p* < 0.001). Similarly, **Table 2** shows the significant association between HTLV-1 infection and hypothyroidism before and after adjusting for age, sex, and residential location. The prevalence of hypothyroidism in the HTLV-1 group and control group was 11% and 3.2%, respectively (*p* < 0.001).

**Table 2:** Association between HTLV-1 and hypothyroidism or hyperthyroidism.

	HTLV-1+ N(%)	HTLV-1− N(%)	Crude Odds Ratio (95% CI)	Adjusted* Odds Ratio (95% CI)	*p*
Hyperthyroidism					
No	298 (88.7)	679 (97.7)	5.1 (2.6–10.5)	6.8 (3.5–13)	**<0.0001**
Yes	38 (11.3)	16 (2.3)			
Age Category (years)			0.99 (0.97–1.00)		
<30	1 (2.6)	5 (31.2)		
30–65	17 (44.7)	9 (56)		0.6 (0.2–1.7)	0.355
>65	20 (52.6)	2 (12.5)		0.4 (0.1–1.2)	0.110
Sex					
Female	31 (81.6)	9 (56.2)		0.6(0.3–1.2)	0.181
Male	7 (18.4)	7 (43.7)			
Residence					
Centre littoral	27 (71.0)	16 (100)		0.7 (0.5–1.0)	0.043
Other	11 (28.9)	0(0)			
Hypothyroidism					
No	299 (89)	673 (96.8)	3.8(2.1–6.8)	3.24 (1.8–6.0)	**<0.0001**
Yes	37 (11)	22(3,2)			
Age Category (years)			1.01(1.00–1.03)		
<30	1 (2.7)	2 (9.1)			
30–65	9 (24.3)	12 (54.5)		0.9 (0.3–1.2)	0.895
>65	28 (75.7)	8 (36.4)		1.8 (0.5–6.2)	0.359
Sex					
Female	29 (78.4)	8 (36.6)		1.0 (0.6–1.9)	0.181
Male	8 (21.6)	14 (63.6)			
Residence					
Centre littoral	24 (64.9)	18 (81.8)		1.0 (0.8–1.3)	0.043
Other	13 (35.1)	4 (18.2)			

*Adjustment using a logistic regression model with age, sex, and residence area as independent variables.

Values in bold are significant values p<0.05.

In women, we found a highly significant difference in hyperthyroidism prevalence: 11.89% in the HTLV-1 group and 1.85% in the control group (*p* < 0.001). Similarly, the prevalence of hypothyroidism was significantly higher in women with HTLV-1 infection (12.87%) than that in the controls (2.11%) (*p* < 0.0001). The prevalence of hypothyroidism and hyperthyroidism in men with HTLV-1 infection was 7.34% and 4.59%, respectively, whereas in the control group, it was 4.43% and 4.59%, respectively, and no significant difference was observed.

In the low-TSH group, there was one case associated with HAM/TSP and one case with ATL, and in the high-SH group, there were two cases associated with HAM/TSP and one case with ATL.

### Risk difference between persons with and without HTLV-1 infection

The risk difference between patients with and without HTLV-1 infection was 8.4% for hyperthyroidism and 5.5% for hypothyroidism, as assessed by inverse probability weighting.

## Discussion

After adjusting for age, sex, and residential location, there were strong associations between HTLV-1 infection and both hyperthyroidism and hypothyroidism.

To date, no baseline estimate of the prevalence of dysthyroidism in French Guiana in the general population exists. The prevalence of hyperthyroidism in the control group (2.3%), although not representative of the general population, was comparable to that found in two studies, conducted in Colorado and São Paulo; it was 2.4% for subclinical hyperthyroidism and 0.7% for proven hyperthyroidism (Tunbridge et al., 1977; Canaris et al., 2000). Regarding hypothyroidism, the São Paulo group had a prevalence of 6.5% for subclinical hypothyroidism and 5.7% for confirmed hypothyroidism; however, this included patients treated after thyroid surgery and patients with treatments potentially causing dysthyroidism (Tunbridge et al., 1977).

The first case of HTLV-1-associated autoimmune thyroiditis in patients with tropical spastic paraparesis was reported by Fukazawa et al. in 1991 (Fukazawa et al., 1991). In 1992, Kawai et al. described two cases of Hashimoto thyroiditis and three cases of Graves’ disease in HTLV-1-positive patients with no other known viral involvement (Kawai et al., 1992a; Kawai et al., 1995). Subsequently, a few cases of Graves’ disease associated with uveitis in HTLV-1-infected patients have been described (Kamoi et al., 2022).

Kawai et al. and Mizokami et al. reported a 6.3% and 7.4% prevalence of HTLV-1 antibodies in patients with Hashimoto thyroiditis, respectively, both of which were significantly higher than that in the general population in their provinces (2.2% and 1.4%, respectively) (Kawai et al., 1992a; Mizokami et al., 1994). Akamine et al. showed that patients with ATLL and asymptomatic HTLV-1 carriers had significantly more anti-TPO (anti-thyroperoxidase) and anti-thyroglobulin (anti-TG) antibodies than the control group of seronegative patients, with an expected female predominance in the seronegative group (Akamine et al., 1996). In a large general population-based cohort, Hawkins et al. reported the prevalence of anti-TPO antibodies to be 9.8% in women and 2.8% in men (Hawkins et al., 1980), and conversely, a balanced male/female ratio in the group of HTLV-1-positive patients, in favor of an involvement of HTLV-1 in the autoimmune mechanism in these patients. Among patients with anti-TPO and anti-TG antibodies, the group infected with HTLV-1 had a significantly greater proportion of biological hypothyroidism than the group that was not, but this observation was based on a small sample.

In a sample with a larger number of HTLV-1-infected persons as compared to that in other studies, Mine et al. showed a significant difference in the prevalence of anti-TPO and anti-TG antibodies between young men with HTLV-1 seropositivity and seronegativity, which is in agreement with a link with HTLV-1 (Mine et al., 1996). A positive trend was observed in patients with anti-HTLV-1 antibodies, but no significant difference was observed. Furthermore, tropism of thyroid cells by HTLV-1 has been explored to explain this association. In 1996, Kawai et al. detected viral envelope proteins, viral mRNA, and HTLV-1 DNA in the thyroid follicular epithelial cells of an HTLV-1-infected patient (one of two cases studied) with Hashimoto disease using Southern blot PCR (Kawai et al., 1996). In 1997, Kubonishi et al. detected proviral DNA of HTLV-1 by PCR in the thyroid tissues of a patient infected with HTLV-1 who successively presented with Graves’ disease and uveitis (Kubonishi et al., 1997). This indicated the integration of proviral DNA into the patient’s DNA, suggesting a prior infection of the tissue by the retrovirus. This hypothesis was also supported by experiments conducted by Matsuda et al. in 2005 (Matsuda et al., 2005). The detection of HTLV-1 in thyroid lesions and the experimental demonstration of tropism of HTLV-1 in thyroid cells reinforce the hypothesis of a link between this virus and the development of autoimmune thyroiditis in certain patients. The presence of HAM/TSP or ATL was not more frequently associated with dysthyroidism. This is consistent with the Kawai study where the number of HTLV1 patients with HAM/TSP and Hashimoto’s thyroiditis was not significantly greater than in asymptomatic HTLV1 carriers (Kawai et al., 1992b). HTLV-1 viral loads were unavailable and prospective studies should evaluate the link between viral load levels and dysthyroidism.

Viruses other than HTLV-1 have been studied for their role in thyroid dysfunction. Most studies have focused on demonstrating a link between autoimmunity and viruses. Thus, adolescents with congenital rubella were more frequently found to have anti-TPO and anti-TG antibodies and showed a greater proportion of thyroid dysfunction than the control group (Clarke et al., 1984). Antibodies against the Epstein–Barr virus were found to be significantly more prevalent in patients with autoimmune thyroiditis than in those in the control group (Vrbikova et al., 1996). A study conducted in children with Hashimoto thyroiditis showed significantly more parvovirus B19 DNA in the blood, indicating a recent infection, compared to the control group, as well as a negative correlation between the time of thyroiditis evolution and probability of finding viral DNA in the blood (Lehmann et al., 2008). Anti-thyroid antibodies were found in patients with chronic HCV infection or those with positive anti-HCV antibodies in 2% to 48% of cases, and the prevalence of subclinical hypothyroidism varied between 2% and 9% in studies (Antonelli et al., 2006). The presence of these viruses in the thyroid has been demonstrated, suggesting their involvement in disease development (Ziring et al., 1977; Mori et al., 2007). Overall, several viruses and mechanisms may affect thyroid function in different ways.

The pathophysiological mechanism of thyroid damage by HTLV-1 is still unknown, but several hypotheses drawing from knowledge about better known viruses, such as HCV, can be put forward. There is a strong genetic influence and heritability in the development of autoimmune dysthyroidism (Shukla et al., 2018). It has been described as the activation of lymphocytes by lymphotropic viruses (Vargas-Uricoechea, 2023). One hypothesis is that some viruses could interact with susceptibility genes in the immune regulatory systems (HLA-DR, CTLA-4, CD40, and PTPN22) and thyroid-specific genes (Thyroglobulin, TSHR) and trigger dysthyroidism through epigenetic modifications (Tomer and Davies, 1993; Jacobson and Tomer, 2007; Hasham and Tomer, 2012). These genes stimulate lymphocyte proliferation and differentiation, and represent an essential immunomodulatory component for thyroid follicular cells and the antigen-presenting cell. Few studies have so far analyzed these gene modulators in relation to the pathophysiological mechanisms of HTLV-related thyroiditis. However, a study carried out in 2022 shows that CTLA4 is not involved in the pathogenesis of HTLV-1-related thyroiditis (Tomoyose et al., 2002). Another hypothetical mechanism for dysthyroidism caused by the hepatitis C virus would be molecular mimicry where shared partial sequences of amino acid segments with the antigens of the thyroid tissue lead affect thyroid function (Cuan-Baltazar and Soto-Vega, 2020). Certain HCV viral envelope proteins can be recognized at the thyroid level, such as the E2 glycoprotein, which can bind to CD81 receptors expressed on thyroid cells and thus induce a cascade of signaling pathways leading to the release of IL-8, a highly proinflammatory cytokine (Akeno et al., 2008; Vargas-Uricoechea, 2023). A recent study in 2020 found that HCV infection of thyrocytes resulted in the production of the chemokine CXCL-8 and the pro-inflammatory cytokines tumor necrosis factor alpha (TNF-α) and a decrease in the expression of the thyroid peroxidase and thyroglobulin genes and an increase in the expression of the deiodinase 2 gene (Hammerstad et al., 2020).

These hypothetical mechanisms could also be involved in the physiopathology of the thyroid disturbances observed with HTLV-1. However, the above hypotheses drawn from an analogy with a very different virus require specific testing. HIV, a retrovirus such as HTLV-1, has been occasionally associated with thyroid dysfunction (notably linked to antiretroviral therapy) but does not seem to offer particular insights to explain our findings. More generally, the role of HTLV-1 infection in the context of autoimmune diseases has been increasingly reported notably for rheumatoid arthritis, systemic lupus erythematosus, and Sjögren’s syndrome. Molecular mimicry has been the main suspected mechanism; however, HTLV-1-promoted altered cytokine release is critical to tolerance rupture. Hence, HTLV-1-induced changes in regulatory CD4 T-cell molecules activity affects the homeostasis of cytokines such as IFN-γ, TNF-α, TGF-β, and IL-10, which disrupts the balance in anti-inflammatory and inflammatory responses, resulting in the loss of tolerance and the development of autoimmunity (Quaresma et al., 2016). The present report suggests that the above list of HTLV-1-associated autoimmune diseases may need to be incremented with thyroid autoimmune diseases. Future studies should systematically test this hypothesis.

For HTLV-1, although the present results support prior studies, the detailed mechanisms remain hypothetical and require further prospective studies and meta-analyses to improve our understanding.

The present study has some limitations. This cross-sectional study of a population of inpatients or outpatients presumably overestimated the baseline prevalence of dysthyroidism in the general population. The risk-difference calculations imply causal relationships that could not be proven given our study design. Nevertheless, this association has been described in different populations, a replication that suggests that this was not just a chance finding and that, in our endemic region, the impact of HTLV-1 on dysthyroidism may be substantial. Thus, it may require adaptations in the standard explorations conducted in patients with dysthyroidism, who should be tested for HTLV-1, and in those with HTLV-1 infections, who should undergo TSH measurements during follow-up. The selection of matched controls was difficult in the older age groups, which distorted the specific analyses of dysthyroidism by sex and age.

The hypothesis of autoimmunity could not be explored because of the reasonably low number of antibodies found in these patients (8/69); therefore, further prospective studies should investigate dysthyroidism in detail.

## Conclusions

Our findings revealed a strong association between HTLV-1 infection and biological abnormalities in TSH levels. Further prospective studies are required to explore the autoimmunity hypothesis. In practice, given our observations and previous work, we propose the conduction of a systematic TSH assay for all patients with positive HTLV-1 serologies, initially and during their follow-up. Furthermore, we propose that patients with dysthyroidism in HTLV-1-endemic regions should be tested for HTLV-1 infection. The screening of persons with HTLV-1 for dysthyroidism is potentially important in the medium and long term because it would allow an early management and control of the broad multisystemic effects of thyroid dysfunction, notably cardiac and bone morbidities for hyperthyroidism.

## Data availability statement

The original contributions presented in the study are included in the article/supplementary material. Further inquiries can be directed to the corresponding author.

## Ethics statement

The studies involving human participants were reviewed and approved by Ethics Committee and the Institutional Review Board of French Guiana. The patients/participants provided their written informed consent to participate in this study.

## Author contributions

Conceptualization: JD, MD, MN, NS; Data curation: JD, MD, NH, HA JMA, KDA, AN, FS, MN, NS; Formal analysis: MN, NS; Investigation: JD, NH, JMA, AN, NS; Methodology: JD, MN, NS; Project administration: JD, MD, HA, FS, MN, NS; Resources: JD, MD, NH, HA, JMA, KDA, AN, FS, NS; Statistical analysis JD, MN, NS; Supervision: MN, NS; Validation: all authors; Visualization: all authors; Manuscript writing: JD, MD, MN, NS.
